# Regulation of Cell Cycle Progression by Growth Factor-Induced Cell Signaling

**DOI:** 10.3390/cells10123327

**Published:** 2021-11-26

**Authors:** Zhixiang Wang

**Affiliations:** Department of Medical Genetics, Faculty of Medicine and Dentistry, University of Alberta, Edmonton, AB T6G2H7, Canada; zhixiang.wang@ualberta.ca

**Keywords:** cell cycle, growth factors, receptor tyrosine kinases, G1 phase, S phase, G2 phase, M phase, Ras/Erk, PI3K/Akt

## Abstract

The cell cycle is the series of events that take place in a cell, which drives it to divide and produce two new daughter cells. The typical cell cycle in eukaryotes is composed of the following phases: G1, S, G2, and M phase. Cell cycle progression is mediated by cyclin-dependent kinases (Cdks) and their regulatory cyclin subunits. However, the driving force of cell cycle progression is growth factor-initiated signaling pathways that control the activity of various Cdk–cyclin complexes. While the mechanism underlying the role of growth factor signaling in G1 phase of cell cycle progression has been largely revealed due to early extensive research, little is known regarding the function and mechanism of growth factor signaling in regulating other phases of the cell cycle, including S, G2, and M phase. In this review, we briefly discuss the process of cell cycle progression through various phases, and we focus on the role of signaling pathways activated by growth factors and their receptor (mostly receptor tyrosine kinases) in regulating cell cycle progression through various phases.

## 1. Introduction

**Figure 1:**
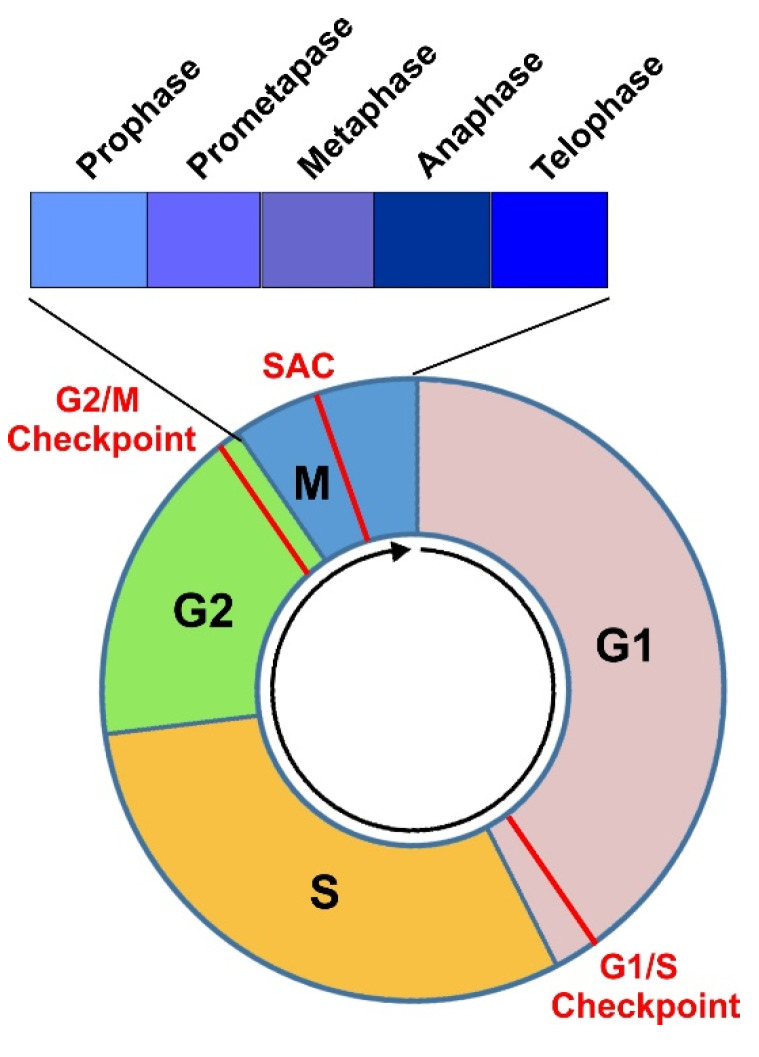
Diagram to illustrate a complete cell cycle progression through four cell cycle phases (G1, S, G2, and M) and three major checkpoints (G1/S, G2/M, and SAC). M phase is further divided into Prophase, Prometaphase, Metaphase, Anaphase, and Telophase.

The cell cycle, or the cell division cycle, is the series of events that take place in a cell that drive it to divide and produce two new daughter cells. The typical cell cycle in eukaryotes is composed of four phases including the G1, S, G2, and M phase. G1, S, and G2 together are called interphase. M phase is comprised of mitosis, in which the cell’s nucleus divides, and cytokinesis, in which the cell’s cytoplasm divides to form two daughter cells. Mitosis and cytokinesis are tightly coupled together. Mitosis is further divided into five subphases including prophase, prometaphase, metaphase, anaphase, and telophase (Figure 1). Each phase of cell cycle progression is reliant on the proper completion of the previous cell cycle phase. A cell could also exit from cell cycle to enter G0 phase, a state of quiescence [1,2].

Cell cycle progression is mediated by cyclin-dependent kinases (Cdks) and their regulatory cyclin subunits. Cdks, such as Cdk4/6, Cdk2, and Cdk1 (also known as Cdc2) are serine/threonine kinases with a wide variety of substrates. Cdks are activated mainly by binding to their cyclin partners, whose expressions rise and fall throughout the cell cycle to mediate the temporal activation of each Cdks. Various cell cycle checkpoints exist to ensure that critical processes are engaged prior to progression to the next phase. There are three major cell cycle checkpoints, including the G1/S checkpoint (also referred as restriction point), the G2/M DNA damage checkpoint, and the spindle assembly checkpoint (SAC) [3,4,5].

Growth factors (GFs) are a group of proteins stimulating the growth of specific tissues. GF plays important roles in the regulation of cell division that drives cell proliferation. Each GF binds to a specific cell–surface receptor. A specific group of GF receptors possesses tyrosine kinases activity and is termed as receptor tyrosine kinases (RTKs). RTK plays most important roles in cell cycle regulation. RTKs are classified into 20 families. The most studied RTKs in terms of cell cycle include epidermal growth factor (EGF) receptor (EGFR) family, insulin receptor family, platelet-derived growth factor (PDGF) receptor (PDGFR) family, and nerve growth factor (NGF) receptor (NGFR). GFs drive cell cycle by activating RTKs and downstream signaling pathways, which regulates cyclin-Cdk complexes [6,7].

In this review, we will briefly discuss the process of cell cycle progression through various phases and will focus on the role of GF/RTK-activated signaling cascades in regulating cell cycle.

## 2. Early History of Cell Cycle Discovery

**Figure 2:**
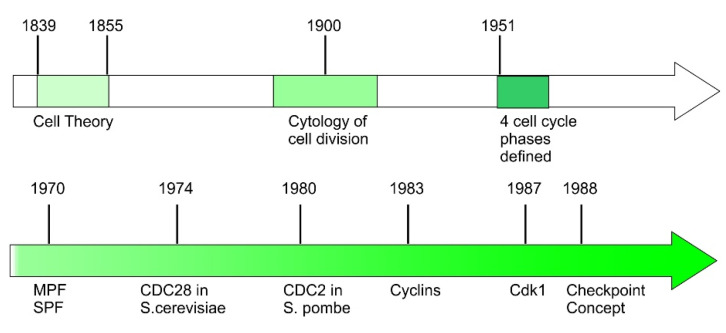
Timeline of major discoveries in the early cell cycle research.

Cell theory was developed in the middle of the 19th century. This theory has three main components: (1) Every living organism is composed of one or more cells; (2) cells are the basic unit of life for all living organisms, and (3) cells only arise from pre-existing cells. While the first two components were the contribution of Theodor Schwann and Matthias Jakob Schleiden, the last is the contribution of German scientist and physician Rudolf Virchow. His discovery that all cells arise from pre-existing cells is the start point of cell cycle research [8,9,10] (Figure 2).

At the turn of the 19th century to 20th century, the cell cycle has been the subject of intense study. The cytology of cell division is described in great detail by microscopists and embryologists, however, the underlying mechanisms driving cell division are mostly unknown. In the late 1970s and 1980s, the advancement of modern molecular biology provided means and knowledge to study the molecular mechanisms regulating cell cycle. Cell biologists, biochemists, and geneticists joined forces and demonstrated that the basic processes and control mechanisms of cell cycle are universal in eukaryotes.

In the late 19th century, early light microscopic studies recognized that cell division follows mitosis, during which cells condensed their chromosomes. Based on his observations of cell division in various stages, German biologist and a founder of cytogenetics Walther Flemming identified the sequence of chromosome movements in mitosis. Flemming’s discovery was proven correct decades later by the study of live dividing cells [11]. However, the only observable morphological changes outside of mitosis is the growth of the cell size. Interphase remained a black box and recognized as one phase until the discovery that DNA synthesis occurs only in a short period during interphase [12]. This discovery split interphase into three phases: This DNA synthesis period is termed as S phase, the gap between mitosis and S phase is termed G1 phase, and the gap between S and M phases is termed as G2 phase [13].

Following the recognition of four major cell cycle states G1, S, G2, and M, the focus of cell cycle study shifted to understand the transition between these phases. A major task is to identify the factors driving the transition. In the early 1970s, by fusing cells at different stages of the cell cycle, it was shown that late G2 or M phase cells contained an M phase-promoting factor (MPF) capable of accelerating the onset of mitosis in early G2 cells [14]. It was further shown that S phase cells contains an S phase-promoting factor (SPF) in nuclei, which is able to accelerate S phase [14,15].

While there is no biochemical method available to purify either MPF or SPF at the time, genetic studies of cell cycle related genes are fruitful. At the end of the 1960s, Leland Hartwell realized the possibility of using genetic methods to study cell cycles. He established budding yeast *Saccharomyces cerevisiae* as a highly suitable model system to study cell cycles. In an elegant series of experiments in 1970–1971, he used the temperature sensitive lethal mutants of *S. cerevisiae* to isolate yeast cells with mutated genes, controlling the cell cycle. By this approach, he successfully identified more than one hundred genes which specifically involved in cell cycle control. Among these genes are genes encoding SPF and MPF. Hartwell named these genes Cdc-genes (cell division cycle genes) [16,17,18]. One particularly important gene identified is Cdc28, which controls the first step of cell cycle progression in G1 phase and was also known as “start”.

In the middle of the 1970s, Paul Nurse followed Hartwell’s approach to study cell cycle regulation with similar genetic methods but using fission yeast *Schizosaccharomyces pombe* as a model system. Through this research, Paul Nurse discovered the gene Cdc2 in fission yeast. Cdc2 is identical to Cdc28 identified in budding yeast. Nurse found that Cdc2 had a key function in the control of transition from G2 to mitosis during cell cycle [19]. In 1987, Nurse isolated the human version of Cdc2 gene, Cdk1. Cdk1 encodes a protein called cyclin-dependent kinase (Cdk). They found that phosphorylation status of the mammalian Cdc2 protein (p34Cdc2) is closely related to cell cycle progression. It is phosphorylated when cells are stimulated to enter the cell cycle in G1 phase, but dephosphorylated when cells go to quiescence [20,21]. Based on these findings, half a dozen different Cdk molecules have been found in humans.

In the early 1980s, Tim Hunt discovered the first cyclin molecule by studying sea urchins, Arbacia. There are eight very rapid cell divisions during the cleavage in embryos of the sea urchin. To sustain these cell divisions, the continual protein synthesis is required. Hunt found that one protein is always destroyed each time the cells divide. This protein was named cyclin as the level of the protein vary periodically during the cell cycle [22]. In the following years, more cyclins were identified in various species by Hunt and other groups. Moreover, it was discovered that the cyclins bind to the Cdk molecules to regulate the Cdk activity and determine the substrate specificity of Cdks [23].

Another important concept introduced during this period is “Checkpoint”. In the late 1980s, by studying the sensitivity of yeast cells to irradiation, Hartwell developed concept of checkpoint [24,25] (Figure 2). He observed that the cell cycle is arrested at certain point when DNA is damaged. This cell cycle checkpoint concept is then expanded as surveillance mechanisms used by the cells to check the integrity, fidelity, and the sequences of the major cell cycle events. The events being monitored include cell size growth, DNA replication, and integrity, and the accurate chromosome segregation [3].

The historical contribution of Leland H. Hartwell, Paul M. Nurse, and R. Timothy (Tim) Hunt earned them 2001 Nobel Prize in Physiology or Medicine for their discovery of “key regulators of the cell cycle”.

## 3. Cell Cycle Progression through Various Phases

The cell cycle consists of G1, S, G2, and M phases. In G1 phase, the cell grows and becomes larger. The cell enters S phase when it reaches a certain size. S phase is the period for DNA-synthesis, during which the cell duplicates its DNA. In the following G2 phase, the cell monitors the completion of DNA-replication and prepares for mitosis. Chromosome segregation and cell division are completed in M phase. The proper cell cycle progression ensures that each of the two daughter cells receives identical chromosome from parent cell. After cell division, the cell cycle is completed, and the cells are back in G1 phase. The duration of the cell cycle varies between 10 and 30 h in most mammalian cells. Cells in the G1 can exit from the cell cycle and enter G0 phase, a state of quiescence.

**Figure 3:**
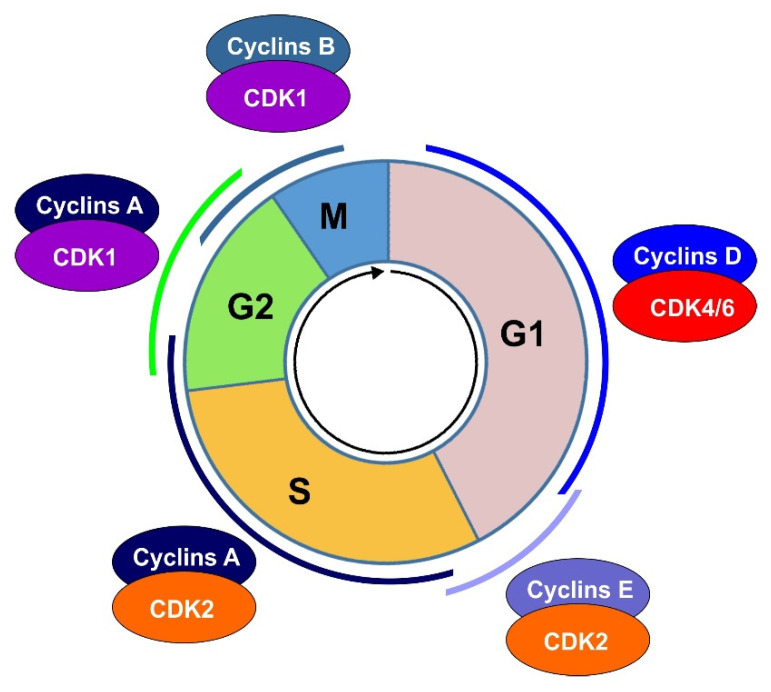
Regulation of cell cycle progression by Cdks and Cyclins.

Cell cycle progression is mainly driven and regulated by two classes of proteins, Cdks and cyclins [26] (Figure 3). In yeast, while several Cdks are expressed, including Cdk1, PHO85, and Kin28, only Cdk1 directly regulates cell cycle progression. Cdk1 is equivalent to p34Cdc2 in *S. pombe* and p34Cdc28 in *S cerevisiae*. By associating with different cell-cycle stage-specific cyclins Cdk1 regulates diverse cell cycle transitions including G1 to S transition and G2 to M transition. The roles of PHO85 and Kin28 in cell-cycle regulation are indirect [26,27]. Higher organisms possess many yeast Cdk1 functional homologues. These functional homologues are phase-specific Cdks. Each phase-specific Cdk acts in a specific cell phase to perform the function of Cdk1 in yeast. Approximately 20 Cdk-related proteins are discovered, which leads to the concept that cell cycle events in higher eukaryotic cells are regulated by complex combinations of Cdks and cyclins in various cell cycle phases. For Cdk/cyclin complexes, cyclins confer substrate specificity and determine the regulatory consequence of the substrates such as activation, inactivation, and localization. Based on this hypothesis, the classical model of cell cycle regulation is established through extensive research in eukaryotic cells.

According to this model, Cdk4 and/or Cdk6 form complexes with D-type cyclins, which activates Cdk4/6 and initiates phosphorylation of the retinoblastoma protein (Rb) family in early G1 phase [28,29]. Rb phosphorylation stimulates the release of transcription factor E2F, which then stimulates the transcription of early E2F responsive genes required for the progression of the cell cycle [30,31]. Early E2F responsive genes include A- and E-type cyclins [28,32]. In the late G1 phase, cyclin E binds to and activate Cdk2, which leads to the full Rb phosphorylation and the further activation of E2F mediated transcription [28,29]. Together, the above events drive the passage of the cell through the restriction point at the boundary of the G1/S phase and initiate the S phase. At the onset of the S phase, A-type cyclins are synthesized and form complex with Cdk2, which phosphorylates proteins involved in DNA replication and drive the cell progression to G2 phase [33,34]. At the late G2 phase, Cdk1/cyclin A is formed and activated, which is required for the G2/M transition and the initiation of prophase [35]. Finally, Cdk1/cyclin B complexes are formed in M phase and drive the completion of mitosis [36] (Figure 3).

### 3.1. G1 Phase

Cells enter G1 either from the preceding M phase or from G0 phase. The transition of cells between G0 and G1 phase is determined by extracellular mitogenic signals [37,38]. G1 phase is the growth phase. The biosynthetic activities of the cell are slowed down considerably in M phase; however, it resumes at a high rate in G1 phase. In G1 phase, the cells synthesize many proteins, amplify organelles including ribosomes and mitochondria, and grow in size. The duration of cell cycle phases varies considerably in different types of cells. For a typical proliferating human cell, if we assume the total cycle time is 24 h, the duration of G1 phase is approximately 11 h, S phase duration last 8 h, G2 phase last 4 h, and the duration of M phase is approximately 1 h.

During G1 phase, diverse signals, including environmental cues, stress, and metabolic cues intervene to influence cell’s developmental program. These signals are integrated and interpreted by the cells. Based on these inputs, the cell decides whether to self-renew, differentiate, or die; however, to enter S phase for starting its renewal, all cells must fulfill one essential requirement: activation of Cdks [38,39].

### 3.2. S Phase

S phase is marked by DNA synthesis. In S phase, each chromosome consists of two sister chromatids following replication to double the amount of DNA. However, S phase also marked with low activities of gene expression and protein synthesis. A noticeable exception is the production of histone. Most histones are produced in the S phase [40].

It is suggested that an intra-S phase checkpoint exists to control S phase progression. Intra-S phase checkpoint turns off Cdk2 in response to DNA damage and other replication stress, which blocks origin firing to avoid replication of damaged DNA [41]. S phase to G2 phase transition is regulated by the active checkpoint kinase ATR (ataxia-telangiectasia and Rad3-related) [42].

### 3.3. G2 Phase

The cell enters G2 phase after successful completion of S phase. G2 phase ends with the onset of mitosis. The major task of cells in G2 phase is to prepare itself for mitosis. G2 phase is marked by significant protein/lipid synthesis and cell growth [43]. While it is known that protein synthesis inhibitor arrests cells at G2 phase, a recent study suggests that this may be due to the inhibition of p38, and the protein synthesis is not absolutely required for mitosis entry [44]. Interestingly, some cell types, including certain cancer cells and *Xenopus* embryos, lack the G2 phase. Cell cycle proceeds directly from S phase to M phase. It is hypothesized that cell size controls the growth in G2 phase, however, this is only demonstrated in fission yeast [45]. Another process that occurs during G2 phase is to repair DNA double-strand breaks. During and after DNA replication, DNA double-strand breaks accumulate in the cell and need to be repaired before cell can move to pass G2/M checkpoint [24,46,47].

### 3.4. Mitosis and Cytokinesis

M phase is comprised of mitosis, in which the cell’s nucleus divides, and cytokinesis, in which the cell’s cytoplasm divides to form two daughter cells. Mitosis is further divided into prophase, prometaphase, metaphase, anaphase, and telophase (Figure 1).

Prophase is characterized with chromatin/chromosome condensation, centrosome separation, and nuclear membrane breakdown. The migration of centrosome to two opposite poles is important for the later formation of the bipolar mitotic spindle apparatus. A recent detailed study shows that the interphase organization is rapidly lost in prophase by a condensin-dependent manner [48]. Observations with a microscope indicate that chromosomes become recognizable as linearly organized structures in early prophase [49]. Sister chromatids are mixed in early prophase, but they are separated in late prophase. Each chromatid is shown as an array of loops radiating from an axial core that contains topoisomerase II alpha and condensin complexes [50]. The rise of cyclin B-Cdk1 activity is a defining molecular event of prophase [51].

Prometaphase starts from the nuclear envelope breakdown, which marks the end of prophase, ends when chromosome alignment at the spindle equator completes, which defines the beginning of metaphase. For faithful chromosome segregation, it is essential to establish a metaphase plate in which all chromosomes aligned at the cell equator attach to mitotic spindle microtubules. The achievement of this configuration depends on the precise coordination of several mitotic events including nuclear envelope breakdown, connection between chromosome kinetochores, and microtubules of the mitotic spindle assembly, and the congression of all chromosomes to the spindle equator. A kinetochore is a disc-shaped protein structure in duplicated chromatids [52]. During prometaphase the chromatids shorten and become thicker [49] and ultimately form fully condensed metaphase chromosomes [53].

Metaphase starts when the duplicated chromosomes are aligned along the metaphase plate in the middle of the cell. During metaphase, the sister chromatids are pulled back and forth by the kinetochore microtubules until they align along the equatorial plane. The chromosome segregation process is monitored by SAC pathway to ensures that all kinetochores are attached to microtubules of the opposite poles before segregation proceeds after metaphase-to-anaphase transition. Once all the chromosomes are properly aligned and the kinetochores are correctly attached, the cohesion between sister chromatids is dissolved, leading to the migration of the separated chromatids towards opposite sides of the cell by the pulling force of spindle microtubules. The cell now enters the anaphase [54].

Anaphase involves two mechanistically distinct steps, the shortening of kinetochore microtubules and the spindle elongation in the midzone. The shortening of kinetochore microtubules causes the migration of each chromatid towards its respective pole. The disjointed sister chromatids are further separated through spindle elongation in the midzone. These two steps may be temporally divided in some organisms while occurring simultaneously in other organisms. These two steps are called anaphase A and anaphase B, respectively [55]. In human mitotic cells, anaphase B usually starts 30–50 s later than the start of the anaphase A [46,56]. During anaphase, the spindle elongates 8 µm and additional 3 µm in telophase [46,55].

Telophase follows anaphase and starts at the onset of the chromosome recondensation and the nuclear envelope reformation [47]. During telophase the duplicated chromosomes in the nucleus of a parent cell separate into two identical daughter cells. A nuclear membrane forms around each set of chromosomes to divide the nuclear DNA from the cytoplasm. Simultaneously, the chromosome decondensation begins [55].

Cytokinesis results the physical separation of the cytoplasm of a mother cell into two daughter cells [57,58]. The segregation of chromosomes and cytoplasm needs to be tightly coordinated to generate offspring with the right complement of chromosomes [59]. Cell cytokinesis is initiated in anaphase, when lower Cdk1 activity causes the reorganization of the mitotic spindle and the stabilization of microtubules. The assembly of the central spindle is the key early event, which provides the template for the midbody and contributes to division plane specification. The division plane is positioned between the two sets of segregated chromosomes. The precise position of the plane is critical to prevent segregation errors. Cytokinetic furrow ingression of the attached plasma membrane is then initiated by the contraction of the actomyosin ring, which partitions the cytoplasm into two domains of emerging daughter cells. The last step of cytokinesis is abscission [60]. Abscission is the physical separation of the plasma membrane of the two daughter cells. During abscission, cells remove the cytoskeletal structures from the intercellular bridge, followed by constriction of the cell cortex, and finally the division of the plasma membrane [61,62].

## 4. The Regulation of Cell Cycle by GF-Initiated Signaling Pathways

GFs play vital role in driving cell proliferation by activating RTK and the downstream signaling cascades. The aberrant activity of these signaling cascades frequently leads to cancer [63,64]. Cell proliferation can only be realized through cell division and early studies have demonstrated that GF stimulation is the driving force of cell cycle initiation and progression. However, after extensive research in the late 1990s and early 2000s, little research directly studies the role of GF/RTK in the regulation of cell cycle. While the role of GF receptor in G1 phase has been well studied, very little is known regarding their role in other phases of cell cycle.

**Figure 4:**
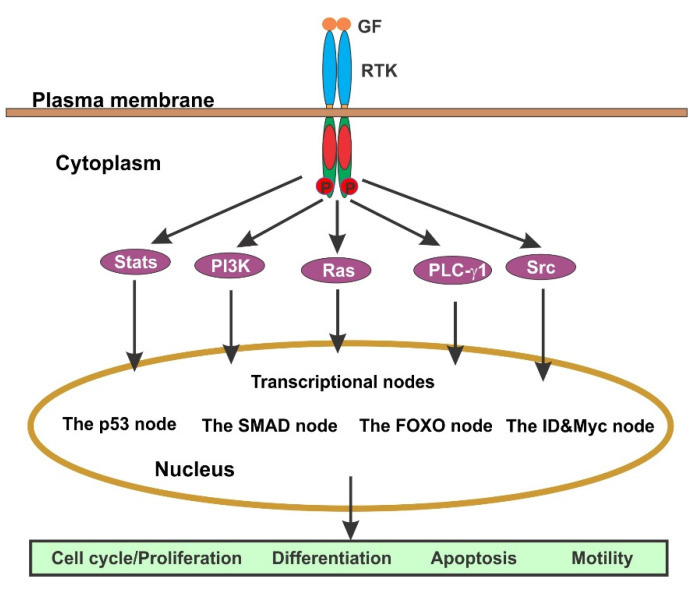
Diagram to illustrate signaling pathways initiated by GF through their membrane receptors, mostly RTKs.

GFs regulate diverse functions of the cells, and the effects of most GFs are mediated by RTKs (Figure 4). Signal transduction starts when a GF binds to its receptor at the cell surface, which stimulates the dimerization of RTK and the activation of its kinase. Two monomers of the RTK dimer phosphorylate each other to fully activate RTK and the phosphorylated Tyr residues in the C-terminus of the receptor become the binding sites for recruiting multiple downstream signaling molecules. The formation of the RTK-signaling protein complex initiates the activation of multiple signaling pathways including Ras/Erk, PI3K/Akt, Src/Jak/Stat, and PLC-γ1. These signaling pathways interact each other to form a signaling network. Signals from various signaling pathways are eventually integrated at the level of transcription. There are several major transcriptional nodes: (1) The p53 node mediates cytostasis and apoptosis in response to high level of mitogenic signals or DNA damage; (2) The SMAD node mediate the expression of cytostatic (arrest of cell growth and multiplication) and apoptotic factors in response to TGF-β; (3) The FOXO node also mediates cytostasis and apoptosis, but in response to oxidative stress and starvation; and (4) ID and Myc node that suppress Cdk inhibitors to favor cell proliferation. By controlling the transcription of specific genes, GF-initiated cell signaling regulates diverse cellular functions including cell migration, cell survival, cell cycle progression, and differentiation (Figure 4) [7,64].

### 4.1. EGFR-Mediated Signaling Pathways

As a prototypical receptor of all RTKs, EGFR signaling network has been extensively studied and very well understood [7,64]. Here, we are using EGFR as an example to illustrate the signaling pathways of GF/RTK. Following its identification in the 1970s [65], EGFR is shown to possess intrinsic kinase activity. The full-length receptor is cloned in 1984 [66]. EGFR is a single polypeptide chain transmembrane glycoprotein. The following is a brief description of the two major signaling pathways activated by EGFR that is most relevant to cell cycle progression.

#### 4.1.1. Ras/Erk Pathway

**Figure 5:**
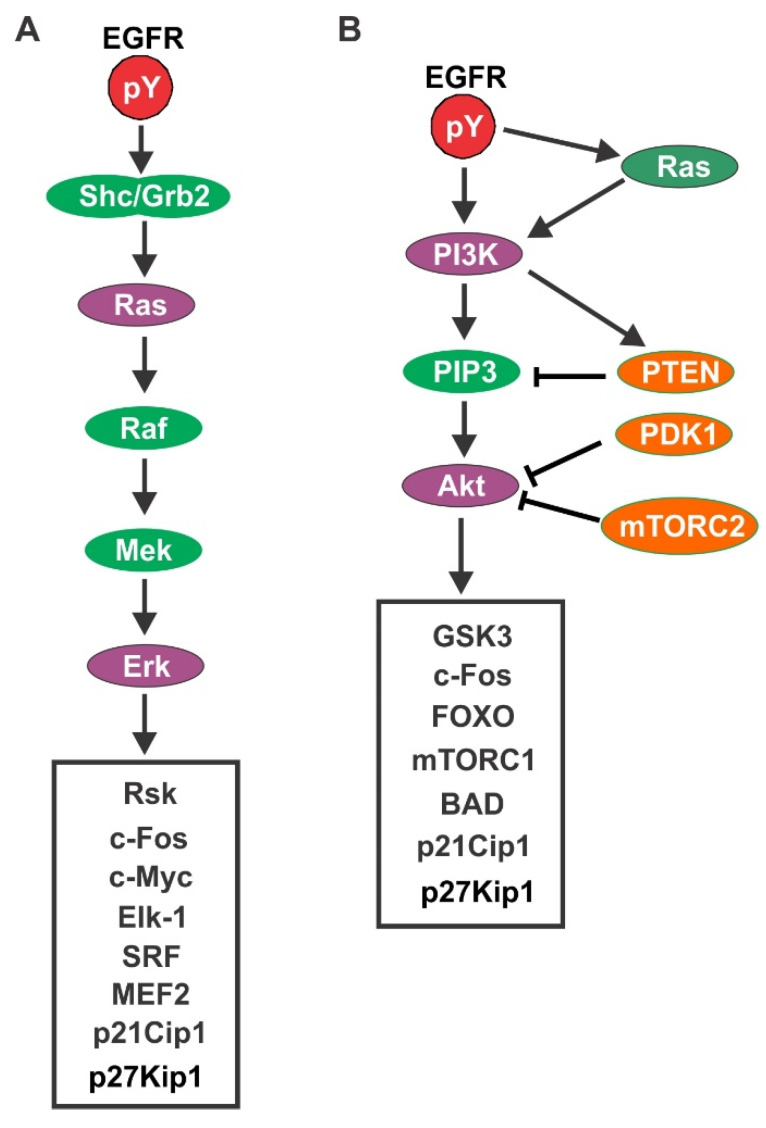
Activation of Ras/Erk (**A**) and PI3K/Akt (**B**) signaling pathways downstream of EGFR.

A major signaling pathway downstream of EGFR is the Ras/Erk pathway. The phosphorylated EGFR interacts with SHC/Grb2, which recruits Sos to cell membrane to activate Ras. Activated Ras then stimulates the activation of Raf. Mitogen-activated protein kinase (MEK) is phosphorylated and activated by Raf, which activates Erk. Erk activation stimulates the activation and nuclear translocation of RSK. In the nucleus, RSK stimulates the activation of transcription factors including c-Fos and SRF. On the other hand, following its activation, Erk also translocates into the nucleus where it stimulates c-Fos and Elk1 (Figure 5A) [67,68,69,70,71,72,73].

#### 4.1.2. PI3K/Akt Pathway

EGFR stimulates PI3K either by binding to its p85 subunit directly or indirectly by activating Ras [74,75] (Figure 5B). The function of PI3K is to generate phosphatidylinositol-3,4,5-trisphosphate (PIP3) by phosphorylating phosphatidylinositol-4,5-bisphosphate (PIP2). However, this process is reversed by the phosphatase and tensin homologue deleted on chromosome 10 (PTEN). PTEN acts as a direct antagonist of PI3K and provides important negative control over the PI3K pathway. It is also reported that PI3K directly interacts with PTEN to stimulate PTEN activity [76,77]. PIP3 interacts with pleckstrin homology (PH) domains to recruit PH-domain containing proteins to the plasma membrane. Among the recruited PH-domain containing protein is the serine threonine kinase Akt. In the plasma membrane, phosphoinositide-dependent kinase 1 (PDK-1) phosphorylates Akt Thr 308 to partially activate Akt. The following phosphorylation of Akt in Ser 473 by the rapamycin complex 2 (mTORC2) results the full activation of Akt. Activated Akt activates multiple downstream substrates, including the forkhead box O transcription factors (FoxO), the BCL2-associated agonist of cell death (BAD), and glycogen synthase kinase 3 (GSK3), to promote cell cycle entry and cell survival. Akt also activates the small G-protein ras homologue enriched in the brain (Rheb), leading to the activation of mTORC1. mTORC1 phosphorylates the eukaryotic translation initiation factor 4E binding protein 1 (4EBP1) and the p70S6 kinase (S6K1), which promotes protein translation and protects the cell from apoptosis [78,79,80].

### 4.2. GF-Activated Ras/Erk Pathway Activation in Cell Cycle Progression

An earlier study indicates that Erk is rapidly phosphorylated in response to GFs including EGF and PDGF [81]. The first evidence regarding the role of Erk in cell proliferation comes from the Erk-inhibition experiments. Inhibition of Erk by various means block the GF-induced cell proliferation, which indicates that GF activation of p42mapk and p44mapk is an absolute requirement for triggering the proliferative response [82]. However, Erk1/2 activation is not sufficient to drive cells into S phase [83,84,85].

**Figure 6:**
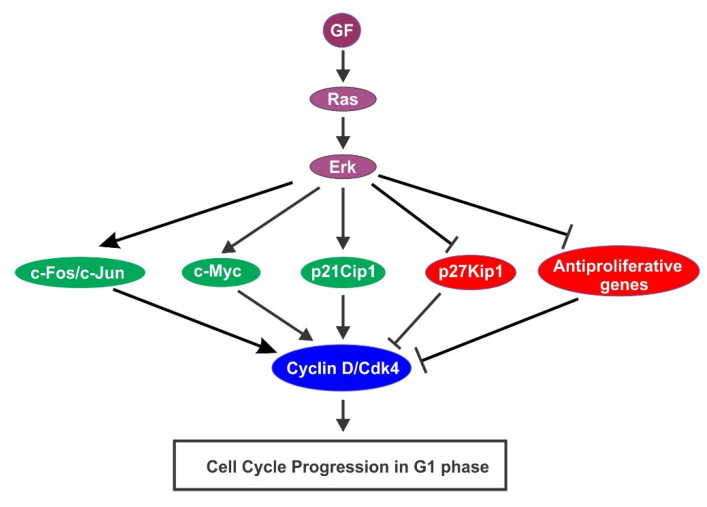
Regulation of cell cycle progression in G1 phase by GF-induced activation of Ras/Erk signaling pathway.

Accumulated evidence indicates that Erk activation promotes G1 progression with multiple mechanisms [86] (Figure 6). One mechanism is by induction of cyclin D and assembly of cyclin D–Cdk4 complexes. Expression of activated Ras in different cell types is sufficient to induce the accumulation of cyclin D1 [87,88,89]. As a downstream signaling protein of Ras, Erk activation is both necessary and sufficient for transcriptional induction of the Cyclin D1 gene [90]. In order for cells to enter S phase, sustained Erk activation is essential to maintain the level of cyclin D1 in G1 phase [91]. The precise mechanism that connects Erk signaling to Cyclin D1 transcription is not clear. However, it is known that the Cyclin D1 promoter contains a functional AP-1 binding site. It is also shown that Erk signaling stimulates the expression of c-Fos and c-Jun, the critical AP-1 components in response to GFs [91,92]. Sustained Erk activation also stabilizes AP-1 proteins by stimulating the phosphorylation of their C-terminal residues [86]. The Erk pathway also regulates the cellular cyclin D1 expression level through the post-transcriptional regulation [86,93]. There is also evidence to support the role of Erk in the induction of cyclin D2 and cyclin D3 [94,95].

The second mechanism is to stabilize transcription factor c-Myc. C-Myc regulates cell cycle progression and apoptosis [96]. Activation of Erk1 by GF strongly enhances the stability of c-Myc protein by direct phosphorylating c-Myc at Ser 62 [97]. C-Myc regulates many proteins that function directly in cell cycle control. These proteins include Cdk4 [98], cyclin D2 [99], Cdc25A [100], and p21 [99]. It is shown that both Erk activation and c-Myc expression are required to drive cells from G0 to late G1 phase [85].

Another mechanism is through the regulation of p21Cip1 and p27Kip1 expression. While p21Cip1 was initially thought to be a cyclin-dependent kinase inhibitor (CKI), further study indicates that p21Cip1 plays multiple roles in regulating Cdk activity. p21Cip1 interact with both Cdk4–cyclin D and Cdk6–cyclin D complexes under physiological conditions, which induces their kinase activation from early G1 to middle S phase [28]. In response to GFs p21Cip1 is transiently accumulated in early G1 phase by an Erk-dependent and p53-independent mechanism [101,102]. A transient activation of Erk is sufficient to induce the expression of p21Cip1 [103], which contributes to the stabilization of cyclin D/Cdk4 complexes in G1 [104]. On the other hand, p21Cip1 also inhibits the activation of Cdk2–cyclin A and Cdk2–cyclin E complexes from late G1 to S phase [105]. Thus, p21Cip1 regulates cell cycle progression by controlling the activation of various Cdks.

Ras/Erk signaling pathway also mediates the GF-induced downregulation of p27Kip1 [84,106,107,108]. It is possible that Erk1/2 downregulates p27Kip1 by stimulating the activation of cyclin D/Cdk4/6 complexes, which induces the degradation of p27Kip1 at the G1/S transition. It is also suggested that Erk signaling downregulates p27Kip1 expression by a Skp2- and Cdk2-independent mechanism [109]. Finally, Erk signaling also contributes indirectly to p27Kip1 regulation by stimulating the synthesis of autocrine GFs [86].

The fourth mechanism is to downregulate antiproliferative genes. As revealed by a gene profiling analysis, 173 antiproliferative genes are downregulated by an Erk-dependent mechanism during G1 phase [110]. It is further shown that to maintain decreased expression levels of antiproliferative genes, continuous activation of Erk throughout G1 is required.

While the activation of the Ras–Erk pathway stimulates cell cycle progression in G1 phase in general, it is interesting to note that some studies suggest that too strong Erk activation results reversible or permanent cell cycle arrest [86] by stimulating the expression of the Cdk inhibitor p21Cip1 [111,112,113,114].

### 4.3. GF-Activated PI3K/Akt Pathway Activation in Cell Cycle Progression

**Figure 7:**
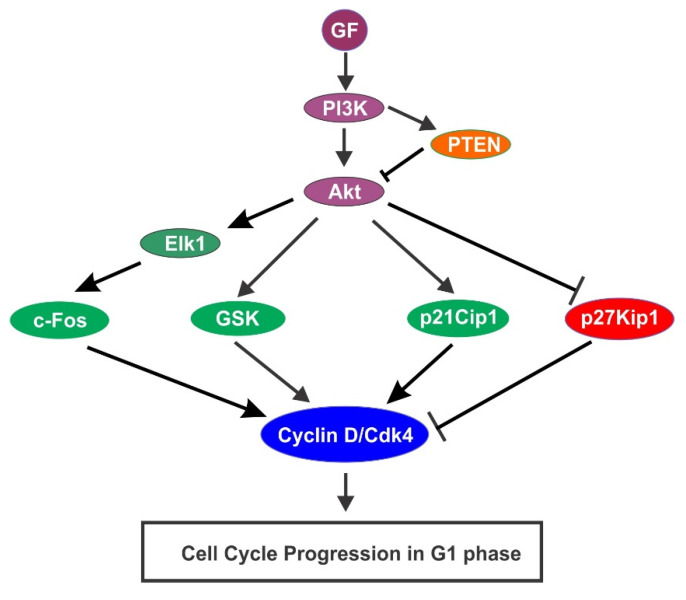
Regulation of cell cycle progression in G1 phase by GF-induced activation of PI3K/Akt signaling pathway.

GFs also regulate cell cycle progression through the activation of PI3K/Akt pathway. It was shown in the early 1990s that PI3K mediated the mitogenic signals of PDGFR [115,116]. In the following years, the role of PI3K in mediating PDGF-induced cell cycle progression were revealed [117,118]. The activation of Akt by PI3K or inhibition by PTEN has been shown to play critical role in GF-induced cell cycle progression [119,120,121]. These studies firmly established roles of PI3K in GF induced cell cycle progression. Extensive research during this period also revealed various mechanisms by which PI3K/Akt pathway regulate in GF-induced cell cycle progression (Figure 7).

Stimulation of p21Cip1 is an important mechanism underlying PI3K/Akt regulation of cell cycle progression. The PI3K/Akt signaling is required for the accumulation of p21Cip1 in human ovarian carcinoma cells. While expression of a constitutively active mutant Akt increases the expression of p21Cip1, expression of dominant negative Akt decreases p21Cip1 expression [122]. Further research about the mechanisms underlying the PI3K/Akt pathway in regulating p21Cip1 expression indicates that Akt directly phosphorylates T145 and S146 near the c-terminus of p21Cip1. These phosphorylations stimulate DNA synthesis and Cdk activity, increasing cellular proliferation [123]. Akt also phosphorylates p21Cip1 at S146, which enhances p21Cip1 protein stability and significantly prolongs the half-life of p21Cip1. High level of p21Cip1 induces assembly and activation of p21Cip1/Cdk4/cyclin D and p21Cip1/Cdk6/cyclin D complexes [124].

The inhibition of p27Kip1 is another mechanism. The PI3K/Akt pathway decreases the expression of p27KIP1. Expression of dominant negative Akt caused transcriptional induction of p27Kip1 in mesenchymal cells, inhibiting both Cdk2 activity and DNA synthesis [125]. It is shown simultaneously by three groups that Akt directly phosphorylates p27Kip1 on T157, which leads to the retention of p27Kip1 in the cytoplasm. Cytoplasm-localized p27Kip1 cannot bind to nuclear Cdk2, thus cannot inhibit Cdk-2 [126,127,128].

The third mechanism is to stimulate cyclin D1 by inhibiting GSK-3. GSK-3 is constitutively active in unstimulated cells and phosphorylates numerous proteins including cyclin D, which triggers the degradation of Cyclin D through the ubiquitin-dependent proteolysis pathway, thus maintains cyclin D in an inactive state by reducing its expression level. Phosphorylation of GSK-3 by PI3K/Akt pathway causes the inactivation of GSK-3, thus promoting the accumulation and activation of cyclin D [129,130,131,132].

The fourth mechanism is the regulation of c-Fos. Expression of a constitutively active PI3K p110 mutant in NIH-3T3 cells induced c-Fos transcription [125]. Moreover, expression of a dominant negative Akt mutant rat mesenchymal cells decreases c-Fos transcription. As discussed above, c-Fos transcription stimulates cell proliferation. It is further shown that PI3K/Akt-induced c-Fos transcription is mediated by Elk-1 [116].

It is notable that the role of PI3K/Akt in the regulation of the cell cycle is heavily influenced by PTEN [133]. As a lipid phosphatase, PTEN antagonizes the function of phosphoinositide 3-kinase (PI3K) by converting phosphatidylinositol-3, 4, 5-trisphosphate (PIP3) to phosphatidylinositol-4, 5-biphosphate (PIP2) [134]. Thus, PTEN inactivates Akt and suppresses cell cycle progression [133].

### 4.4. Co-Regulation of Cell Cycle Progression by PI3K/Akt Pathway and Ras/Erk Pathway

**Figure 8:**
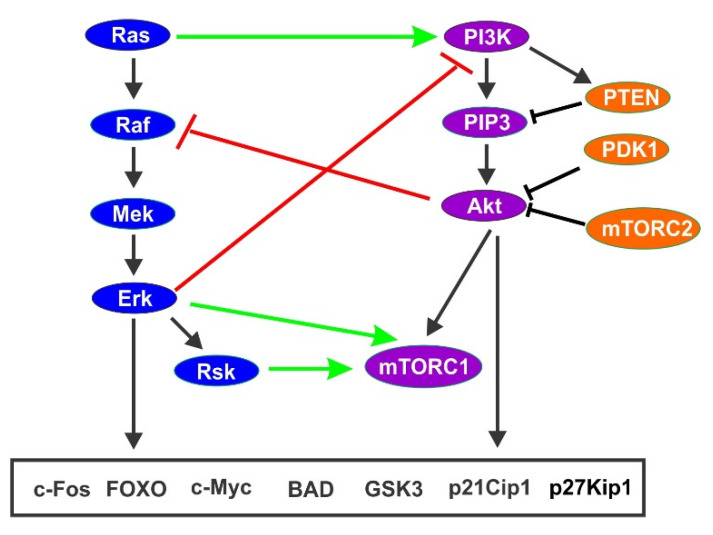
Interplay between Ras/Erk pathway and PI3K/Akt pathway in terms of cell cycle regulation. Green arrow indicates stimulation and red line indicates inhibition. Bottom box listed cell cycle related protein regulated by both signaling pathways.

Upon GF stimulation, the PI3K/Akt and Ras/Erk pathways co-regulate cell cycle progression by interacting closely [135]. Ras/Erk pathway and PI3K/Akt pathway interact at multiple point to coordinately regulate cell cycle progression. Many crosstalk mechanisms have been revealed between these two pathways. These include cross-activation, cross-inhibition, and pathway convergence on substrates [136] (Figure 8).

First, Ras itself is an upstream activator of PI3K, thus activation of Ras leads to the activation of both Erk and PI3K [137]. In addition, Raf may activate Akt in hematopoietic cells [131].

The second mechanism is through mutual inhibition. Ras/Erk signaling may negatively regulate PI3K/Akt signaling and vice versa [138,139]. It is known that activated Erk phosphorylates GAB1, which inhibits GAB1-mediated recruitment of PI3K to the EGFR for activation [140]. It is shown that Runx2 expression relieves Erk-mediated negative regulation of EGFR and Akt [141]. It is also reported that MEK suppresses PI3K signaling by promoting membrane localization of the phosphatase PTEN [142]. Cross-inhibition between Akt and Raf by IGF1 stimulation is also reported [143]. Akt inhibits Raf1 by phosphorylating Raf N-terminal inhibitory sites, which leads to the inhibition of Erk [144,145].

The most striking feature of the crosstalk is that both Ras/Erk pathway and PI3K/Akt pathway regulate same proteins involved in the regulation of cell cycle. As discussed above, both pathways regulate p21Cip1, p27Kip1, GSK-3, Cyclin D, and c-Fos, which control cell cycle progression [86,131].

## 5. Two Waves of GF Signaling Drives Cell Cycle in G1 Phase

Cell cycle progression is controlled at multiple stages. During the early time of cell cycle research, a key regulatory step identified was the restriction point in cultured cells. GF is needed to drive the cell cycle to pass the restriction point, and once the restriction point is passed, GF is no longer required for the continuation of cell cycle progression [146]. What we know now is that the restriction point is G1/S checkpoint, which represents commitment to subsequent DNA replication (S phase). As discussed above, at a restriction point a signaling activity threshold must be reached to allow the continuation of the cell cycle progression. Mitogenic signals generated from GFs/RTKs are sufficient to drive the cells pass the restriction point.

**Figure 9:**
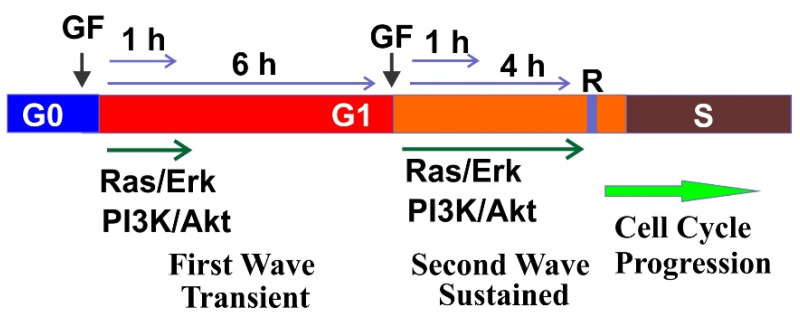
Two waves of GF-induced cell signaling in G1 phase drive the cell cycle progression to pass the restriction point (R).

The earliest studies of the restriction point in mammalian cells indicated that different GFs function differently in regulating cell cycle [147,148]. PDGF was shown as a competence factor, and EGF and IGF-I were defined as progression factors. However, later research shows that different GF may function similarly in driving cell cycle progression [85,149]. As revealed by live imaging in a study using dual-reporter cell model, when compared with serum-free medium, insulin-like GF-I (IGF-I) enhances cell cycle entry by more than 5-fold. Similar results are also obtained when GFs such as EGF, PDGF-AA, and PDGF-BB are used [150]. Another early observation is that the continuous presence of the GF for at least 6–8 h is required for cell cycle progression to pass restrict point [151,152]. Later research finds that the continuous presence of GF could be substituted with two short pulses of GF, one at early G1 phase and one at late G1 phase [6,153]. It is revealed that even with the continuous presence of GFs, the downstream signaling proteins including Ras/Erk and PI3K show two waves of activation (Figure 9).

In the continuous presence of GFs, Ras shows two waves of activation, an early transient activation and a more sustained activation approximately 4 h later [154]. Interestingly, it has been shown by earlier study that Ras activity is needed for cell cycle progression in two distinct times during the G1 phase [155,156], which corresponds to the two waves of Ras activation. In addition to functioning as a molecular switch for re-entry into the cell cycle from the G0 to G1 phase, Ras functions late in G1 to induce p27kip1 downregulation and cell cycle progression to pass the restriction point in response to EGF [157].

Similarly, GF also stimulates two waves of PI3K activation during G1 phase. It is shown that inhibition of PI3K catalytic subunit p110α (but not p110β) several hours after PDGF stimulation suppresses PDGF-dependent DNA synthesis [158]. In the mid-G1 phase, treatment with PI3K inhibitors inhibits GF-dependent cell-cycle progression and the inhibition is due to impaired degradation of p27Kip1 [159]. Indeed, a second, prolonged wave of accumulation of PI3K products is detected 3–7 h after PDGF stimulation [117]. By using an inhibition–rescue approach, this research further shows that, unlike the initial wave, the second wave of PI3K activation is required for PDGF-dependent DNA synthesis.

While prolonged presence of EGF is required to stimulate cell proliferation, we show that two short pulses of EGF are also sufficient to stimulate cell proliferation. We also show that two pulses of endosomal EGFR signaling are sufficient to stimulate cell proliferation [153,160]. The first pulse of EGFR signaling stimulates the exit from G0 into G1 phase. The second pulse, required 4–8 h later, drives the transition of cells from late G1 into S phase [153]. We also show that two waves of PDGFR signaling from endosome are sufficient to drive cell cycle progression [161].

The effects of the two short pulse of GF exposure on cell signaling network is further studied by a comprehensive transcriptomic and proteomic analyses. It is revealed that three processes are responsible for regulating restriction point crossing. In addition to activating essential metabolic enzymes, the first pulse also relieves p53-related restraining processes. The study also shows that the second pulse eliminates the suppressive action of p53 by activating the PI3K/Akt pathway. Finally, the second pulse uses the Erk-EGR1 threshold mechanism to digitize the graded external signal into an all-or-nothing decision-making obligation into the S phase [149]. Recent research shows that Ras signals mainly through Erk in G1, however, it also functions through PI3K/Akt to induce Cyclin D, driving S-phase entry [162].

Together, the findings to date highlight the importance of GF signaling for the progression of the cell cycle. These studies also demonstrated the presence of two action period for GF signaling in G1 phase. The first is in the early stage of G1 phase, which is critical for the G0/G1 transition to commit the cells to enter cell cycle, instead of going into a quiescent state. The second is right before the R-point, which is critical to drive the cells to pass the G1/S checkpoint and to start DNA synthesis.

## 6. GF Signaling in S, G2, and M Phase of Cell Cycle

The study regarding the role of GF-mediated signaling in other cell cycle phases are limited and controversial. The initial studies in the early 1990s to establish the role of GF-mediated cell signaling in cell cycle regulation suggests that GF-activated signaling cascades especially Ras/Erk and PI3K/Akt signaling pathways are required for the cell cycle progression from G1 to S phase by passing through restriction point [86,131]. The role of GF-activated signaling pathways in other phases only began to emerge in the late 1990s to early 2000s.

While it is proposed that GFs only regulate cell cycle progression during G0 to S interval [85], sporadic publications have emerged and shown the involvement of GF-mediated cell signaling in S and G2 phase [163,164,165]. It is shown that the presence of hepatocyte GF in S phase induces G2 delay by sustaining the activation of Erk [164]. On the other hand, it is reported that activation of EGFR by EGF in S phase induces centrosome separation, which promotes mitotic progression and cell survival [166]. The data regarding the role of EGFR activation in G2 phase are also limited and controversial. While all data indicate that EGF stimulates the activation of MEK-Erk signaling pathways [165,167], the findings regarding the effects of activated MEK-Erk pathway are quite different.

It is reported that activation of MEK-Erk signaling pathway by okadaic acid is required for entry into M phase and cell survival [168] and activation of both PI3K/Akt and MEK/Erk pathways by FBS is required for mitotic entry [169]. However, other studies indicate that activation of MEK-Erk signaling pathway by EGFR or phorbol 12-myristate 13-acetate (PMA) in G2 phase delays the M phase entry through p21Cip1 [166]. Activation of Erk signaling by EGF or PMA also induces the G2 phase delay and the blocked exit from G2 checkpoint by destabilizing Cdc25B [170]. By following live cells in which ERK1/2 activity was inhibited through late G2 and mitosis, it is shown that, for a timely entry into mitosis, Erk activity in early G2 is essential. However, Erk activity from late G2 through mitosis does not directly affect cell cycle progression [171].

The role of GF-induced cell signaling in mitosis has been less studied and poorly understood. It is reported that while the level of EGFR expression is the same between M phase and interphase, EGFR-mediated cell signaling pathways is tightly suppressed in M phase as EGF fails to bind to EGFR with high affinity to induce EGFR dimerization [170]. It is reported that EGFR, PLC-γ1, GTPase-activating protein, and Erk2 are less phosphorylated in M phase than in interphase [168]. A further study shows that Cdc2 inhibits EGF-induced Erk activation in M phase [169]. These studies argue that inhibition of GF signaling in M phase shelters the cell from extracellular signals during cell division, which helps the cell to preserve the precious energy needed for mitotic structural changes [168,169,170].

However, we recently showed that in M phase, EGFR is both expressed at the same level and activated to the same level by EGF as in interphase [171]. We further show that, in mitosis, EGFR is phosphorylated at all the major tyrosine residues in the C-terminus to the level similar to that in interphase, suggesting that EGFR is fully activated. However, the fully activated EGFR regulates downstream signaling pathways differently from interphase. It selectively activates some downstream signaling pathways while avoid others. Two major differences include the activation of Akt2, not Akt1, and inability to activate Erk despite of strong activation of Ras [172]. The activation level of other signaling proteins including PLC-γ1, PI3K, Cbl, and Src are all similar between mitosis and interphase. While EGF promotes cell survival in mitosis, it does not alter mitosis progression significantly. The only effect observed is the longer mitosis with the inhibition of EGFR by inhibitor AG1478 [172,173].

By using a cell line that is defective in EGFR downregulation, and thus maintains sustained EGFR signaling, it is shown that EGF-induced activation of EGFR signaling is required in G2 phase to drive the transition of cell cycle from G2 to M phase [174].

A novel method combining quantitative time-lapse fluorescence microscopy and microinjection is developed to examine cell cycle progression without cell synchronization [175,176,177]. By using this method, series research analyzes the role of Ras in the regulation of cyclin D expression and cell cycle progression. The results show that cyclin D1 is induced in a Ras-dependent manner in asynchronous NIH3T3 cells from S to G2 transition during cell cycle. Interestingly, the expression of cyclin D1 is Ras independent during the next G1 phase once induced in G2 phase. It is further shown that the Ras-dependent induction of cyclin D1 in the S/G2 transition is mediated by post-transcriptional mechanisms [178]. While endogenous Ras is active in all cell cycle phases, cyclin D1 is only induced during G2 phase in cycling cells, which indicates that the function of Ras is regulated by cell cycle phase [179]. Constitutively activated mutant Ras accelerates the cell cycle transition through G2/M and renders the G2/M checkpoint and SAC ineffective [180].

The downstream signaling proteins of Ras have also been shown to be involved in the cell cycle phase rather than G0–G1. For example, the MAPK kinase 1 (MAPKK1) activity in synchronized NIH 3T3 cells affects the kinetics of the cell cycle progression through both the G1 and G2 phases. Inhibition of MAPKK1 by dominant negative mutant is also found to delay progression of cells through G2. Moreover, inhibition of MAPKK1 in cells synchronized to S phase arrests the cell in G2 phase, which demonstrates a role for MAPKK1 in G2/M transition [181]. It is interesting to note that some studies suggest that too much Erk activity at the G2/M transition blocks entry in mitosis. Cells lacking VHR arrest at the G1-S and G2-M transitions of the cell cycle, which is dependent on the hyperactivation of Jnk and Erk. Moreover, this arrest is reversed by Jnk and Erk inhibition [182]. Hyperactivation of the Erk pathway due to expression of activated Ras or Raf mutants arrest cell cycle progression by promoting the accumulation of cyclin-dependent kinase inhibitors [86]. Wentilactone B (WB), a tetranorditerpenoid derivative induces G2/M phase arrest in human hepatoma SMMC-7721 cells via the Ras/Raf/Erk and Ras/Raf/JNK signaling pathways [183].

Inhibition of the PI3K/Akt pathway with PTEN or a PI-3 kinase inhibitor results in the cell cycle arrest in G2 phase and overexpression of constitutively active Akt kinase relieves this inhibition [184,185,186]. A PI3K inhibitor shortens the IR-induced G2 arrest [187]. These findings suggest a role of PI3K/Akt pathway G2 phase of cell cycle. Further study suggests that Chk1 kinase mediates the function of Akt in G2 phase. Akt kinase activity increase in G2/M, which coincides with the fall in Chk1 kinase activity [186]. Inhibition of PI3K by inhibitor LY294002 increase Chk1 kinase activity by decreasing Akt activity. Moreover, constitutively active Akt inhibits hydroxyurea-induced activation of Chk1 activation, which relieving DNA damage-induced G2 arrest [186]. Multiple mechanisms underly the ability of Akt to inhibit Chk1. These mechanisms include phosphorylation, ubiquitination, and reduced nuclear localization [188]. It is also reported that blocking PI3K/Akt signaling prolongs progression through S/G2 [189].

By using a Time-Resolved Single-Cell Imaging method, EGF-induced signal processing in individual cells is quantitated over time, which reveals the dynamic contribution of various signaling pathways [190]. It is shown that both PI3K and Erk activity are required for initial cell cycle entry, however, only PI3K activity regulates the duration of S phase. Importantly, if PI3k activity is blocked for 10–20 h after EGF treatment, the durations of S and G2 phase is increased dramatically. A likely underlying mechanism is the phosphorylation and subcellular translocation of Cdk2 by Akt during S phase [190].

## 7. Conclusions

**Figure 10:**
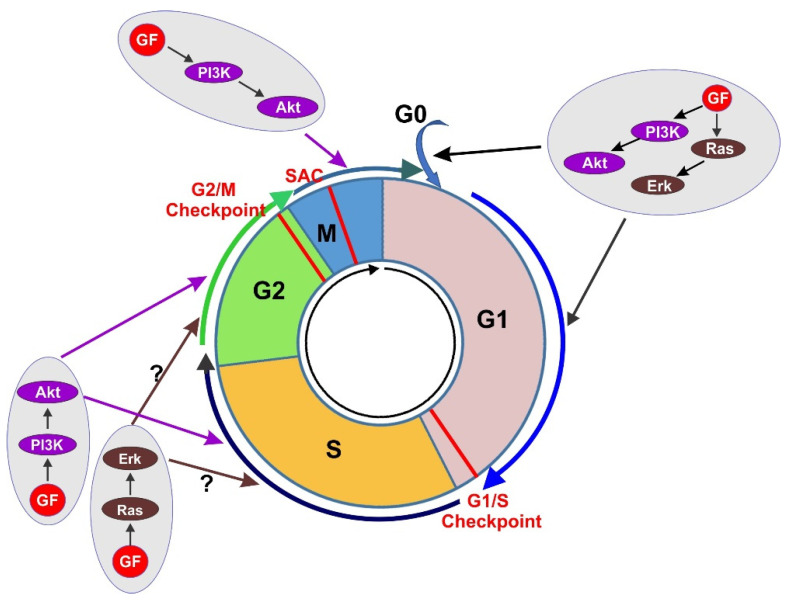
Regulation of cell cycle progression by GF-induced cell signaling in various phases of cell cycle. Question mark indicates that the data are limited and controversial.

Mitogenic signals of GFs are mediated by their transmembrane receptors, mostly RTKs. RTK activation initiates the activation of multiple downstream signaling cascades. Both Ras/Erk signaling pathway and PI3K/Akt signaling pathway play major role in regulating cell cycle progression. While the mechanism underlying the role of GF signaling in G1 phase of cell cycle progression has been largely revealed due to early extensive research, little is known regarding the function and mechanism of GF signaling in regulating other phases of cell cycle including S, G2, and M phase. Accumulated results so far suggest that GF signaling my regulate cell cycle progression throughout the cell cycle, but further research is needed to sustain these findings and to uncover the underlying molecular mechanisms (Figure 10).
